# The Hospital

**Published:** 1917-12-15

**Authors:** 


THE
### INSTITUTIONAL WORKER
**Being a Special Supplement to "The Hospital"**

### OUR BUREAU OF INFORMATION.

**Rules for Correspondents.**

1. Every letter must be accompanied by the coupon to be cut from the back cover (inside page) of THE HOSPITAL, current issue, and must contain the name and address of the correspondent with pseudonym for publication if desired. Replies by post cannot be given save under exceptional circumstances at the Editor's discretion.

2. Letters from Approved Homes in reply to special needs published in the Bureau should state terms and full particulars, and be sent prepaid under cover to the Editor of the Bureau with name written across coupon for identification.

3. Proprietors of Homes which have not yet been entered on the List of Approved Homes, but have spare accommodation likely to suit special needs, are invited to write for an application form for registration. The fee for registration, which includes two announcements of the Home in the Bureau and other privileges, is 10s.

4. All communications to be addressed to the Editor of THE HOSPITAL, 28 Southampton Street, Strand, London, W.C. 2, and marked "Bureau of Information."

### AN ECONOMY CAMPAIGN IN HOSPITALS.

### Professional and Unprofessional Lectures Compared.

AT this time, when so many commodities in daily use in hospitals are with great difficulty obtained in sufficient quantities and cost so much more money than before the war, or than during the earlier stages of the war, it is not surprising that our hospitals are taking steps to survey carefully their resources and to decide how best to conserve them. Last week two metropolitan institutions—the Royal Free Hospital, Gray's Inn Road, W.C., and the National Hospital for the Paralysed and Epileptic, Queen Square, W.C., convened meetings to consider and discuss economies. As the methods of approaching the subject differed in these cases, some description of the proceedings may be helpful to other institutions in choosing plans to deal with this important subject.

AT THE ROYAL FREE HOSPITAL.

To the meeting, which was held in an upper ward on December 5, the authorities had invited Mrs. Peel, one of the lecturers of the Ministry of Food, to give an address. There was a large attendance of the nursing staff and of people connected with the hospital, who listened politely and attentively while the lecturer, during an address lasting forty-five minutes, dealt with the problem of food shortage, comparing our trials with those of the German people, set forth the advantages of voluntary rationing over a ticket system, and showed how greatly some of our Allies have suffered and how little we have suffered. The speech was excellent in tone, and if addressed to an audience of factory workers, let us say, would no doubt have been helpful. Directed, however, to a gathering of hospital folk and others, themselves social workers, mostly educated women, who had had full opportunity through reading their newspapers to gain possession of the same information as that offered to them by the lecturer, her object was not perhaps much advanced until question-time arrived. Then what we suspect was the general feeling of the audience was well expressed in the first question, put by a military nursing sister. She asked, "Will the lecturer kindly give us some practical information as to how best we may in institutions effect economies in the use of food?" Mrs. Peel then rose to the occasion, and offered some useful suggestions. The first was in respect of milk puddings. We strongly suspect that milk (especially rice) puddings, by their too frequent inclusion in meals, are not especially popular amongst the nursing staffs of our hospitals—but that is no reason why they should not be well and economically made. This is Mrs. Peel's method: Soak your cereals for twelve to fourteen hours before using, in just as much water as they can absorb, then boil for ten minutes, and bake gently for three hours. The result is a 10 per cent. to 15 per cent. save in the food value. Another suggestion was with regard to pulses; peas and beans should be soaked for at least twenty-four hours before cooking. Mrs. Peel dwelt upon the value of good cooking (but good cooks are scarce!). There was as much waste through over-roasting as through under-roasting. Further, English people do not sufficiently study soup-making.

AT THE NATIONAL HOSPITAL.

The other economy meeting, on December 2, was a more homely affair. There the chairman, Sir Frederick Macmillan, called the nursing and domestic staffs together and addressed them on some points which seemed to him important at all times, but most essential to consider just now. Being a mere man, Sir Frederick wisely did not enter into the mysteries of cooking, but dealt largely with matters more within his province, such as coal-saving and economy in things involving the use of coal, such as steam, hot water, gas, and electric light. He mentioned the insidious danger of the poker, without the frequent use of which a fire would get along quite nicely. He said that he had heard of gas-rings being left alight because matches were short.

In respect of **washing**, it was suggested, always remembering that cleanliness is next to godliness, that laundry expenses could be reduced if everybody saw to it that a fair amount of use was got out of an article before it returned to the laundry; moreover, laundries being what they are, another point is that the oftener an article is washed the quicker it is worn out.

The use or mis-use of **ward stationery** was mentioned, and the audience were asked never to use an official form for scribbling purposes. In respect of **drugs, chemicals and dressings**, which cost the hospital last year £4,000, it was obviously necessary to be careful to cut down the expense by using just as much material as is necessary.

The Chairman said that the board had before them at their meetings a book showing **breakages of crockery**, etc., together with the names of the persons responsible. He said that the list was looked upon very sadly, and all the board could do was to express disapproval and pay the bills. He hoped that, especially while these articles were so expensive and difficult to replace, all nursing and general staffs would do their best to avoid accidents.

The **expenditure** of the hospital had increased from £18,000 to £30,000 a year, and the income had not expanded accordingly. Sir Frederick concluded by remarking that if every worker at the hospital could manage by some means to save a shilling a week the aggregate amount yearly would be £400.

SOME PRACTICAL HINTS.

These hospital meetings cannot do anything but good. Care must be taken that nurses and others do not get it into their heads that they are being scolded; they must be encouraged to ask questions and to make suggestions, making, in short, a causerie of the affair rather than a formal lecture. Moreover, at the end of the day's work tired people do not want to sit down and listen to a long address, however eloquent. They much prefer, we are sure, to attend a homely gathering in which they can, should they care to do so, take a part.

### Question for December.

### A System of Time-keeping for the Nursing and Domestic Staff.

What is the most satisfactory method of checking the going out and coming in of the nursing and domestic staffs of an institution which has three outlets, none of which it is practicable to keep locked during the day, nor is it possible to keep porters on duty for the purpose?

RULES.

The following rules must be observed:—

1. Contributions must be written on one side of the paper. Brevity and terseness are desirable features. The MSS. must bear the name and address of the sender and be accompanied by coupon to be cut from the back cover (inside page) of the current issue of The Hospital. A pseudonym must be chosen if the name is not to be published.
2. Contributions must be addressed to the Editor of The Hospital, Institutional Worker Supplement, 28 & 29 Southampton Street, Strand, London, W.C. 2, should reach him before the end of the current month, and be marked in the left-hand corner "Facts and Figures."

**A minimum payment of Half-a-Guinea will be made for each published answer.**

### QUESTIONS INVITED.

In connection with our Question Box, we offer every month five shillings each for the two best questions which are sent in for consideration. The questions may be on any institutional subject and concern any department, from the secretary's to the porter's, or the matron's to the domestic staff; the one essential is that they must be practical and deal with points that have a definite relation to hospital or institutional administration, upkeep, finance, or management. Points relating to artificers' work, housekeeping, the laundry, out-patients, and other practical matters will be welcome. It is hoped that thus, when difficult or doubtful points arise in the course of their work, institutional workers will be encouraged to put them in the form of a question and send them to the Editor, The Hospital Bureau of Information, 28 Southampton Street, Strand, W.C. 2, marked "Question Box."

The best questions will be published in due course and our readers will be given the opportunity of answering them. Thus all institutional workers may be able to co-operate with the view of helping the work and smoothing the difficulties of each other. In short, we want the Question Box of the *Institutional Worker* to be freely used by every worker habitually.

### ENQUIRIES AND ANSWERS.

### *WAR MATTERS.*

### Railway Vouchers for Nurses.

The privilege applies to nurses at institutions which accommodate military as well as civil patients. The address of the County Director of the County of London is, Territorial Force Association of the County of London, Duke of York's Headquarters, Chelsea, S.W. 3.—Matron.

### *THE SICK AND IN NEED.*

### West of England Sanatorium.

Owing to the higher prices prevailing for all commodities, the authorities of the Royal West of England Sanatorium, Weston-super-Mare, have been obliged to increase their charges for admission to 31s. per fortnight all the year round, and no patient can stay longer than two weeks. Men and women are taken alternately. Applications should be made to the Honorary Lady Superintendent.—Nina.

### Sanatoria for Children.

Children suffering from consumption are admitted to the Children's Sanatorium, Holt, Norfolk; the Salop Convalescent Home, Baschurch, Shrewsbury; and the Sanatorium for Children, Harpenden, Herts. Write to the Secretary of each institution for full particulars of admission, and at the same time give full information of the case you wish to send.—Joseph.

### Eye Hospitals in Liverpool.

You can obtain two years' training in "eye work" at the Liverpool Eye and Ear Infirmary, Myrtle Street, Liverpool, or St. Paul's Eye Hospital, Oldhall Street, Liverpool. At the former institution applicants are received for training at the age of twenty-one to twenty-four, and the salary given is £10 the first year and £14 the second. The second-named institution receives candidates at nineteen to twenty-four years of age, and the salary is £12 the first year and £14 the second. Laundry and indoor uniform are provided at both institutions.—Inquirer.

### *MISCELLANEOUS.*

### Handbooks for Nurses.

You will find the "Handbook for Nurses," by J. K. Watson, M.D., a very useful help to you. It is published by The Scientific Press, Ltd., 28 and 29 Southampton Street, Strand, W.C. 2, price 5s. 6d. net. A useful book for junior probationers and any who contemplate entering the nursing profession is "First Lines in Nursing," by Miss E. Margaret Fox, price 2s. 6d. net, which is also published by The Scientific Press, Ltd.—Nancy.

### Definition of "Nursing Home."

The term "Nursing Home" in this country implies an institution where it is possible to obtain accommodation for a sick person and the services of a nurse. Medical attention is not, as a general rule, provided, for the obvious reason that these homes depend for their *clientèle* upon surgeons and physicians who require this accommodation and service for their patients, and who continue to treat them after admission. It is in this latter respect that the nursing home differs from a hospital, which is now generally understood to be an institution which undertakes the care and medical treatment of sick persons. In the earlier usage the term was applied to institutions for the support of the aged and infirm, for the support and education of children, as well as for the treatment of the sick. There is no good reason why nursing homes should not be termed hospitals, and, as a matter of fact, it is becoming customary to refer to the most modern of nursing homes as hospitals for paying patients. This has long been the custom in America.—S. L.

### *EMPLOYMENT AND TRAINING.*

### Massage Training.

We suggest that you write to the Secretary of the Incorporated Society of Trained Masseuses, 157 Great Portland Street, London, W., who will advise you as to training-schools for massage in London.—Taffy.

### Special Training in Nervous Diseases.

Candidates are received at the West End Hospital for Diseases of the Nervous System, 73 Welbeck Street, W., for a period of two years' training at the age of (not under) twenty-one. Lectures are given on massage and electrical work, on general nursing and bandaging, and on nervous diseases and their nursing. Salary at the rate of £8 and £10 a year respectively is given. Laundry and part indoor uniform are provided. A three years' course of training can be obtained at the National Hospital for the Paralysed and Epileptic, Queen Square, Bloomsbury, W.C., but candidates must be between twenty-three and thirty-three years of age. Apply to the Matron in each case.—Duncan.

### Midwifery Training in Derby.

You could obtain your midwifery training at the Nightingale Nursing Home, Derby. This institution is the in-patient department of the Royal Derby and Derbyshire Nursing and Sanitary Association. The address is 1 Trinity Street, London Road, Derby. Trained nurses are received for a shorter period of training than the untrained candidate. Lectures are given on midwifery and general nursing, massage, and sick cookery. The fee for trained nurses is £12 12s.—Sherington.

